# In silico validation of RNA-Seq results can identify gene fusions with oncogenic potential in glioblastoma

**DOI:** 10.1038/s41598-022-18608-8

**Published:** 2022-08-24

**Authors:** Ainhoa Hernandez, Ana Maria Muñoz-Mármol, Anna Esteve-Codina, Francesc Alameda, Cristina Carrato, Estela Pineda, Oriol Arpí-Lluciá, Maria Martinez-García, Mar Mallo, Marta Gut, Sonia del Barco, Oscar Gallego, Marc Dabad, Carlos Mesia, Beatriz Bellosillo, Marta Domenech, Noemí Vidal, Iban Aldecoa, Nuria de la Iglesia, Carmen Balana

**Affiliations:** 1 Institut Catala d’Oncologia (ICO) Badalona, Badalona Applied Research Group in Oncology (B-ARGO Group), Institut Investigació Germans Trias I Pujol (IGTP), Carretera Canyet s/n, 08916 Badalona, Spain; 2 Pathology Department, Hospital Universitari Germans Trias I Pujol, Badalona, Spain; 3 CNAG-CRG, Centre for Genomic Regulation, Barcelona Institute of Science and Technology, Universitat Pompeu Fabra (UPF), Barcelona, Spain; 4 Pathology Department, Neuropathology Unit, Hospital del Mar, Institut Hospital del Mar d’Investigacions Mèdiques (IMIM), Barcelona, Spain; 5 Medical Oncology, Hospital Clínic, Translational Genomics and Targeted Therapeutics in Solid Tumors, August Pi I Sunyer Biomedical Research Institute (IDIBAPS), Barcelona, Spain; 6 Cancer Research Program, Institut Hospital del Mar d’Investigacions Mèdiques (IMIM), Barcelona, Spain; 7 Medical Oncology, Hospital del Mar, Barcelona, Spain; 8 Institut de Recerca Contra La Leucèmia Josep Carreras, Badalona, Spain; 9 Medical Oncology, Institut Catala d’Oncologia (ICO) Girona, Hospital Josep Trueta, Girona, Spain; 10 Medical Oncology, Hospital de Sant Pau, Barcelona, Spain; 11 Neuro-Oncology Unit and Medical Oncology Department, Institut Catala d’Oncologia (ICO) Bellvitge, Institut de Investigació Bellvitge (IDIBELL), L’Hospitalet, Barcelona, Spain; 12 Pathology Department, Hospital Universitari de Bellvitge, Bellvitge, Spain; 13 Department of Pathology, Biomedical Diagnostic Centre (CDB) and Neurological Tissue Bank of the Biobank-IDIBAPS, Hospital Clinic, University of Barcelona, Barcelona, Spain; 14 IrsiCaixa AIDS Research Institute, Hospital Universitari Germans Trias I Pujol, Badalona, Spain

**Keywords:** Molecular biology, Cancer, CNS cancer, Neuroscience, Molecular neuroscience

## Abstract

RNA-Sequencing (RNA-Seq) can identify gene fusions in tumors, but not all these fusions have functional consequences. Using multiple data bases, we have performed an in silico analysis of fusions detected by RNA-Seq in tumor samples from 139 newly diagnosed glioblastoma patients to identify in-frame fusions with predictable oncogenic potential. Among 61 samples with fusions, there were 103 different fusions, involving 167 different genes, including 20 known oncogenes or tumor suppressor genes (TSGs), 16 associated with cancer but not oncogenes or TSGs, and 32 not associated with cancer but previously shown to be involved in fusions in gliomas. After selecting in-frame fusions able to produce a protein product and running Oncofuse, we identified 30 fusions with predictable oncogenic potential and classified them into four non-overlapping categories: six previously described in cancer; six involving an oncogene or TSG; four predicted by Oncofuse to have oncogenic potential; and 14 other in-frame fusions. Only 24 patients harbored one or more of these 30 fusions, and only two fusions were present in more than one patient: *FGFR3::TACC3* and *EGFR::SEPTIN14*. This in silico study provides a good starting point for the identification of gene fusions with functional consequences in the pathogenesis or treatment of glioblastoma.

## Introduction

Glioblastoma is the most aggressive primary brain tumor. Standard therapy is surgery followed by radiation therapy with concomitant and adjuvant temozolomide, but median survival remains around 14–16 months^1,2^. Except for the prolonged progression-free—but not overall – survival afforded by bevacizumab, no pharmacological intervention has been able to alter the course of the disease^3,4^. Considering this poor prognosis and lack of effective therapies, it is clearly important to develop novel treatment strategies based on molecular data.

Gene fusions are chimeras of two coding or regulatory DNA sequences. Some result from genomic rearrangements that give rise to a single transcription unit, while others originate by trans-splicing and are only present at the transcript level. Several biological processes contribute to the formation of gene fusions and there are multiple computational tools to analyze them^5^. The increasing importance of gene fusions in solid tumors has recently been recognized due to the emergence of high-throughput technologies, such as RNA-Sequencing (RNA-Seq)^6^. Gene fusions have been described in different tumor types, but most appear not to have functional consequences, although some are involved in the initial steps of tumor development and progression^7,8^.

The first fusion to be identified in glioblastoma was *FIG::ROS1*, in which an intrachromosomal deletion leads to a constitutively active kinase with oncogenic activity^9^. Since then, multiple studies and case reports have described different low-frequency fusions in 30–50% of glioblastomas^10^. The genes most involved in fusions in IDH wild-type glioblastoma are *EGFR* (6–13%), *FGFR3* (3%), *MET* (1–4%) and the *NTRK* gene family (1–2%). All of these genes codify for receptor tyrosine kinases, whose rearrangement leads to oncogenic kinase activation^11^. Several drugs have been approved by the FDA as standard therapy for tumor patients harboring specific gene fusions^12–14^, but glioblastomas are underrepresented because of the low frequency of recurrent fusions. Traditional methods for detecting fusions include Southern blotting, fluorescent in situ hybridization (FISH), and RT-PCR. Next generation sequencing (NGS), including RNA-Seq, can provide a wealth of information on gene expression and chromosomal rearrangements. However, data interpretation is hindered by several constraints: false positives and negatives can confound results; fusions known to be present in healthy tissue must be ruled out as they do not have oncogenic potential; and the translational function of the fusion (driver vs passenger) needs to be identified. Moreover, not all fusions involve genes with a potential or demonstrated role in cancer and not all of them generate in-frame gene fusions, with transcripts that could produce a protein with functional biological effects. Hence, not all fusions are optimal candidates for further validation^15^.

We have examined gene fusions detected by RNA-Seq in a series of newly diagnosed glioblastomas and performed an in silico study to predict their oncogenic potential. We selected fusions with demonstrated or possible oncogenic potential and examined their frequency and their correlation with patient characteristics and outcome, with the aim of identifying frequently recurrent fusions that warranted validation.

## Results

Tumor tissue samples were obtained from 329 of the 432 glioblastoma patients registered in the GLIOCAT project^16^. After multiple RNA extractions from each sample, 357 RNA libraries were prepared and RNA-Seq results were obtained for 151 patient tumor samples. Fusions were assessed with STAR fusion^6^ in 139 formalin-fixed, paraffin-embedded (FFPE) samples obtained at first surgery. Four of these samples had paired fresh-frozen (FF) samples obtained at first surgery and four had FFPE samples obtained at a second surgery performed at the time of recurrence. Data on molecular subtypes^17^ according to the Gene Set Enrichment Analysis (ssGSEA) and Instrinsic Glioma Subtypes (IGS) algorithms were available for 124 samples obtained at first surgery using the GLIOVIS and the R clusterRepro packages^18,19^.

### Gene fusions by initial RNA-Seq in glioblastoma samples

Among the 139 patients with FFPE samples obtained at first surgery, RNA-Seq detected one or more fusions in 61 (43.9%). Table 1 displays the patient characteristics according to the presence or absence of fusions. Fusions were more prevalent in the classical TCGA and the IGS-18 subtypes. Tumors with MGMT methylation had more fusions than those without methylation. Of the 68 tumors with MGMT methylation, 36 (59%) had fusions, while of the 63 tumors without MGMT methylation, only 20 (32.8%) had fusions (*P* = 0.01).

**Table 1:** Characteristics of 139 glioblastoma patients with FFPE tumor samples obtained at first surgery.

	All Patients N = 139 N (%)	Patients without gene fusions by RNA-Seq N = 78 N (%)	Patients with gene fusions by RNA-Seq N = 61 N (%)	p*
Median age, years (range)	63.0 (54.5–70.0)	62.0 (54.0–69.0)	64.0 (55.0–71.0)	0.242
**Age group**				1.000
≤ 65 years	83 (59.7)	47 (60.3)	36 (59.0)	
> 65 years	56 (40.3)	31 (39.7)	25 (41.0)	
**Sex**				0.606
Men	82 (59.0)	48 (61.5)	34 (55.7)	
Women	57 (41.0)	30 (38.5)	27 (44.3)	
**Surgery**				0.429
Unknown	12 (8.63)	7 (8.97)	5 (8.20)	
Gross-total	29 (20.9)	19 (24.4)	10 (16.4)	
Subtotal	83 (59.7)	42 (53.8)	41 (67.2)	
Biopsy	15 (10.8)	10 (12.8)	5 (8.20)	
***MGMT* methylation status**				0.011
Unknown	8 (5.76)	3 (3.85)	5 (8.20)	
Methylated	68 (48.9)	32 (41.0)	36 (59.0)	
Unmethylated	63 (45.3)	43 (55.1)	20 (32.8)	
**G-CIMP**				0.002
Unknown	15 (10.8)	14 (17.9)	1 (1.6)	
No	118 (84.9)	60 (76.9)	58 (95.1)	
Yes	6 (4.32)	4 (5.13)	2 (3.3)	
**TCGA subtype**				< 0.001
Unknown	15 (10.8)	14 (17.9)	1 (1.6)	
Classical	53 (38.1)	22 (28.2)	31 (50.9)	
Mesenchymal	32 (23.0)	24 (30.8)	8 (13.1)	
Proneural	39 (28.1)	18 (23.1)	21 (34.4)	
**IGS_subtype**				0.001
Unknown	15 (10.8)	14 (17.9)	1 (1.64)	
IGS0	6 (4.32)	4 (5.13)	2 (3.28)	
IGS16	2 (1.44)	2 (2.56)	0 (0.00)	
IGS17	12 (8.63)	7 (8.97)	5 (8.20)	
IGS18	68 (48.9)	32 (41.0)	36 (59.0)	
IGS22	4 (2.88)	2 (2.56)	2 (3.28)	
IGS23	18 (12.9)	14 (17.9)	4 (6.56)	
IGS9	14 (10.1)	3 (3.85)	11 (18.0)	
**IDH1 (by IHC)**				0.895
Unknown	12 (8.63)	6 (7.69)	6 (9.84)	
Negative	122 (87.8)	69 (88.5)	53 (86.9)	
Mutated	5 (3.60)	3 (3.85)	2 (3.28)	
**Long survival**				
≤ 30 months	115 (82.7)	67 (85.9)	48 (78.7)	0.374
> 30 months	24 (17.3)	11 (14.1)	13 (21.3)	

*MGMT* O-6-methylguanine-DNA methyltransferase, *TCGA* the cancer genome atlas, *IGS* intrinsic gene expression subtypes, *IHC* immunohistochemistry, *G-CIMP* glioma CpG island methylator phenotype.

*p-value for comparison between patients with and without gene fusions in their tumor samples.

Among the 61 tumor samples with fusions, there were a total of 263 fusions, corresponding to 103 different fusions, with a median of two fusions per sample (range, 1–13). Nine fusions were detected in more than one sample and 101 were detected more than once in the same sample at different breakpoints (Supplementary Table S1 and Supplementary Dataset 1).

**Figure 1:**
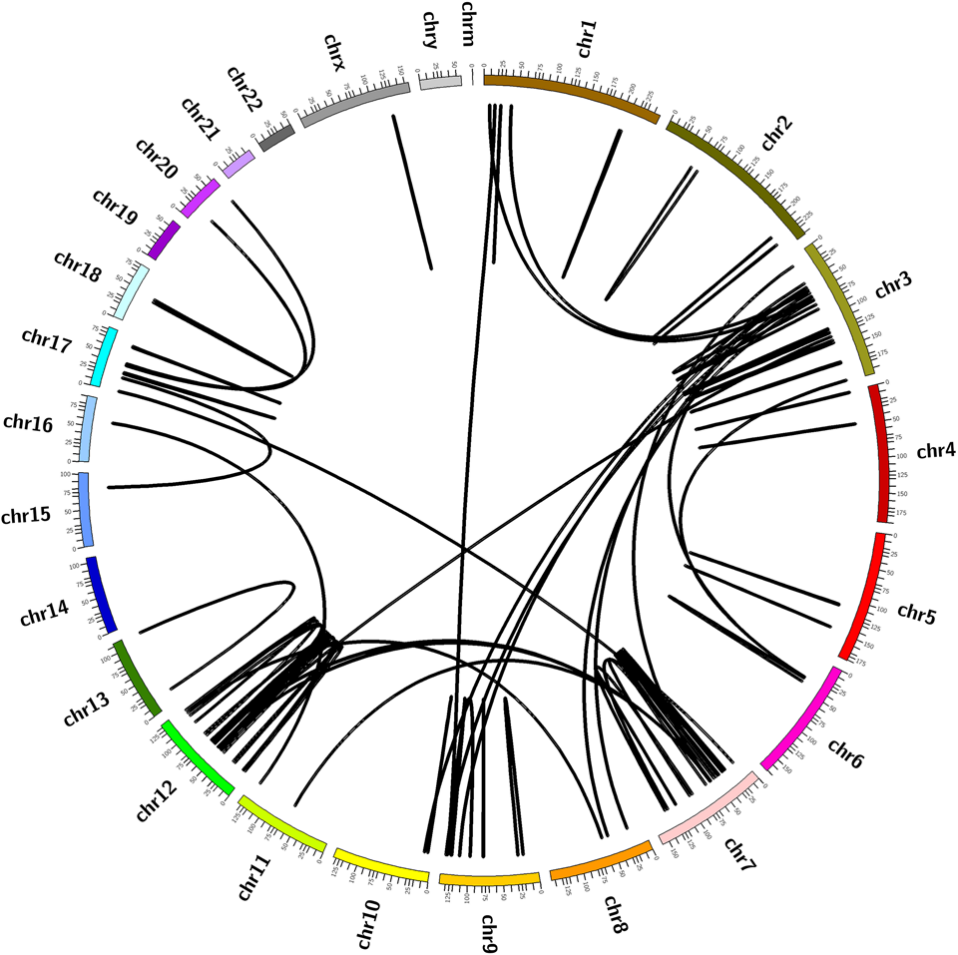
Circos plot showing the chromosomes involved in the fusions detected in this study.

Of the 103 different fusions detected, 79 were intrachromosomal and 24 interchromosomal. The majority of fusions were located at chromosome 12, where there were 40 fusions, 34 of which were intrachromosomal (33% of all fusions). Chromosome 7 had 22 fusions, 15 of which were intrachromosomal (14.6% of all fusions). Chromosomes 3 and 9 had 16 and 8 fusions, respectively (Fig. 1).

Using FusionHub^20^, we classified fusions according to the type of genes they included. The 103 fusions involved 167 different genes (Supplementary Tables S2A and S2B and Supplementary Dataset 1) that can be classified as follows: 1) known oncogenes or tumor suppressor genes (TSGs) (n = 20, 11.9%); 2) genes associated with cancer but not oncogenes or TSGs (n = 16, 9.6%); 3) genes not associated with cancer but involved in fusions in gliomas (n = 32, 19.2%); 4) not associated with cancer and not involved in fusions in gliomas (n = 99, 59.3%) (Supplementary Tables S3A–D, respectively, and Supplementary Dataset 1).

### Selection of fusions with oncogenic potential

**Figure 2:**
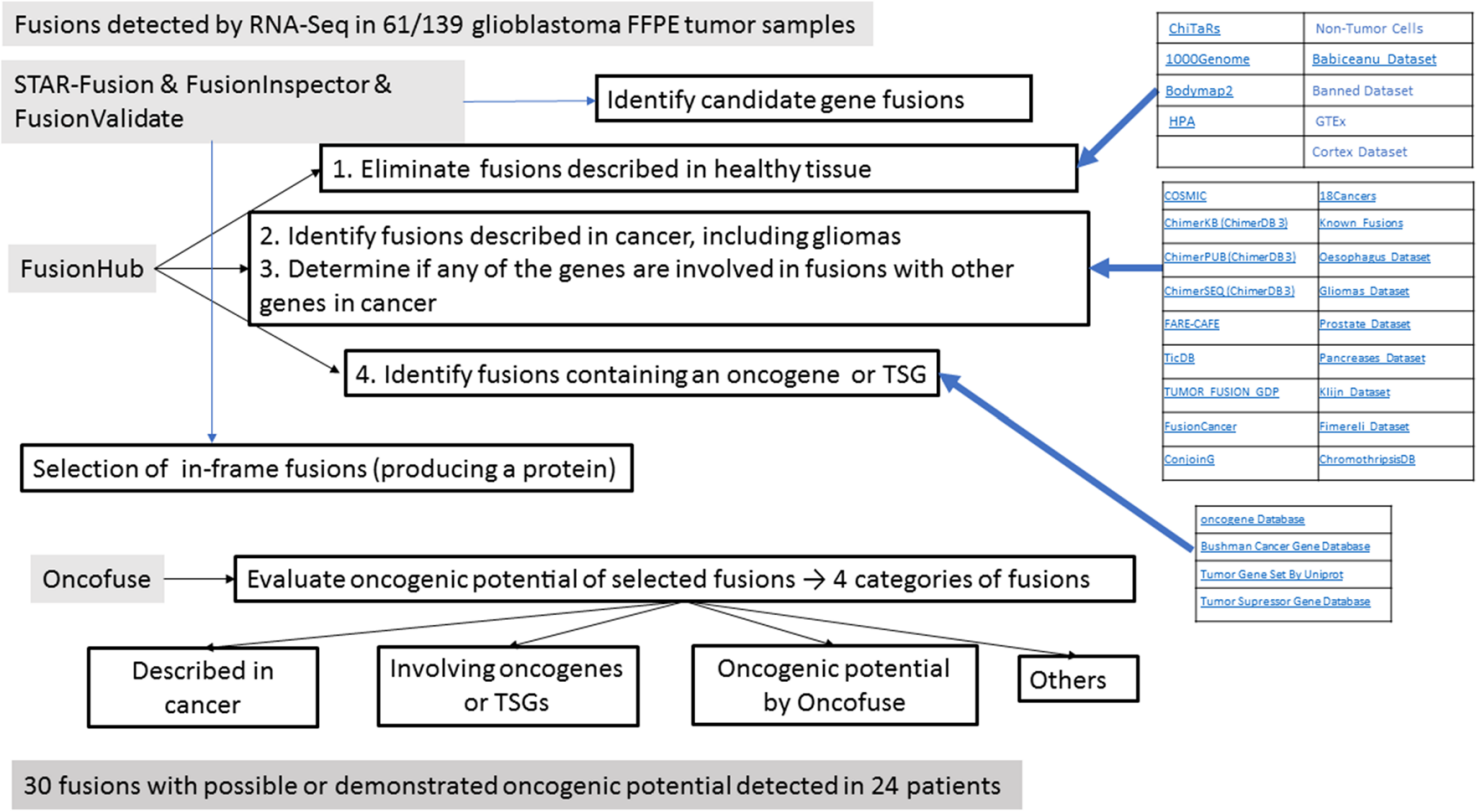
Procedures and data bases used in the present study to select the fusions with oncogenic potential. From the long list of fusions detected by RNA-Seq, we used STAR-Fusion to detect fusion genes and FusionInspector to validate predicted fusions. We then used FusionHub to eliminate fusions previously described in healthy tissue, identify fusions previously described in cancers, and explore whether either gene had been identified as an oncogene or tumor suppressor gene (TSG) or had been associated with cancer. We next used FusionValidate to select only in-frame fusions and finally ran Oncofuse to predict the oncogenic potential of each fusion.

As shown in Fig. 2, we first eliminated six fusions (detected in 45 samples) because they had previously been detected in healthy tissue (Table 2), which would indicate no relevant role in cancer. Of the remaining 97 fusions, ten (detected in 14 samples) had previously been detected in cancers, including gliomas (Table 2) and 21 (detected in 33 samples) had not previously been identified in cancer but included an oncogene or TSG (Table 3). The remaining 66 fusions have not been described in healthy tissue or in cancer and did not include an oncogene or TSG.

**Table 2:** Classification of fusions detected in 61 glioblastoma samples: fusions previously detected in healthy tissue (N = 6) or in cancers (N = 10).

Fusion	Detected in no. samples	Previously described in cancer	Previously described in gliomas	% of all patients included	Type of fusion
**Detected in healthy tissue (N = 45 samples)**					
*KCNMB4::CNOT2*	1	No	No	0.72	In-frame
*NUP214::TMOD1*	1	No	No	0.72	Frame-shifted
*PFKFB3::RP11-563J2.2*	3	No	No	2.16	Unknown
*PID1::DNER*	1	No	No	0.72	In-frame
*RP1-34H18.1::NAV3*	10	No	No	7.2	Unknown
*RP11-444D3.1::SOX5*	29	No	No	21	Unknown
**Detected in cancers (N = 14 samples)**					
*FRS2::KIF5A*	1	Yes	GB	0.72	Unknown
*EGFR::SEPTIN14*	2	Yes	LG & GB	1.44	In-frame
*FGFR3::TACC3*	3	Yes	LG & GB	2.16	In-frame
*CAPZA2::MET*	1	Yes	No	0.72	In-frame
*CLIC4::SRRM1*	2	Yes	No	1.44	In-frame
*DPYSL3::JAKMIP2*	1	Yes	No	0.72	In-frame
*LANCL2::VOPP1*	1	Yes	No	0.72	Unknown
*R3HDM2::AVIL*	1	Yes	No	0.72	Unknown
*RAB3IP::BEST3*	1	Yes	No	0.72	Frame-shifted
*SEC61G::EGFR*	1	Yes	No	0.72	In-frame / Frame-shifted

*LG* low-grade glioma, *GB* glioblastoma.

**Table 3:** Classification of fusions detected in 61 glioblastoma samples: fusions not previously detected in healthy tissue or cancer but involving an oncogene or TSG (N = 21).

Fusion	Detected in no. samples	Left gene			Right gene			Type of fusion
		Oncogene or TSG?	Previously described in cancer?	In fusions with other genes in glioma?	Oncogene or TSG?	Previously described in cancer?	In fusions with other genes in glioma?	
*RP11-384F7.2::LSAMP*	13	No	No	No	Possible TSG	No	LG	Unknown
*GNAQ::CEP78*	1	Oncogene	Yes	No	No	No	No	Frame-shifted
*MALAT1::EGFR*	1	Oncogene	Yes	No	Oncogene or TSG	Yes	HG & LG	Unknown
*MITF::ST18*	1	Oncogene	Yes	No	No	No	No	Unknown
*RERE::PSMD6*	1	Oncogene	Yes	LG	No	No	No	Frame-shifted
*VPS53::VWDE*	1	Possible TSG	No	LG	No	No	No	Frame-shifted
*XRCC5::LINC01614*	1	Possible TSG	No	LG	No	No	No	Unknown
*ABL1::SZRD1*	1	Oncogene or TSG	Yes	No	No	No	No	In-frame / Frame-shifted
*AGAP2::KIF5A*	1	Oncogene or TSG	Yes	GB	No	No	HG & LG	In-frame
*CDK6::RP11-745C15.2*	1	Oncogene or TSG	Yes	No	No	No	No	Unknown
*EGFR::R3HDM2*	1	Oncogene or TSG	Yes	HG & LG	No	No	GB	In-frame
*EGFR::RP11-745C15.2*	1	Oncogene or TSG	Yes	HG & LG	No	No	No	Unknown
*HMGA2::LLPH*	1	Oncogene or TSG	Yes	GB	No	No	No	Frame-shifted
*JAZF1::SEPT7P5*	1	Oncogene or TSG	Yes	HG & LG	No	No	No	Unknown
*STAT3::CFAP61*	1	Oncogene or TSG	Yes	No	No	No	No	Unknown
*USP22::TMC3*	1	Oncogene or TSG	No	No	No	No	LG	In-frame
*BEST3::EGFR*	1	No	No	No	Oncogene or TSG	Yes	GB	Unknown
*C3orf62::PBRM1*	1	No	No	No	Oncogene	Yes	LG	Frame-shifted
*CEP78::GNAQ*	1	No	No	No	Oncogene	Yes	No	In-frame
*CTDSP2::GLI1*	1	No	No	GB & LG	Oncogene or TSG	Yes	HG & LG	In-frame
*SLC35E3::EGFR*	1	No	No	GB	Oncogene or TSG	Yes	HG & LG	Frame-shifted

*TSG* tumor suppressor gene, *LG* low-grade glioma, *HG* high-grade glioma, *GB* glioblastoma.

After eliminating the frame-shifted fusions, verifying the breakpoints with the Integrative Genomics Viewer^21^ and running Oncofuse^22^, we classified the remaining 30 fusions in the four previously established categories: (1) six were previously described in cancer; (2) six were not previously described in cancer but involved an oncogene or TSG; (3) four were predicted by Oncofuse to have oncogenic potential; and (4) 14 were other in-frame fusions that can produce a protein but that have not previously been described in cancer, do not involve an oncogene or TSG, and were not predicted to have oncogenic potential by Oncofuse (Supplementary Dataset 2). These 30 different fusions were considered to have oncogenic potential (Table 4).

**Table 4:** Characteristics of patients with tumors harboring one or more of 30 gene fusions with oncogenic potential.

Tumor samples N = 24	No. fusions detected per sample	Fusions detected in each sample	Patient characteristics								
			Age	Sex	MGMT promoter methylation?	Type of glioma	Survival (months)	G-CIMP?	IDH1 mutations? (by IHC)	TCGA subtype	IGS subtype
AC0340	1	*PID1::DNER*^a^	67	Man	No	Primary	7.62	No	No	Pro	9
AC0346	1	*ACVR1B::SCAF11*^a^	50	Man	Yes	Secondary	30.49	Yes	Yes	Pro	9
AC0365	1	*CNOT2::RBMS2*^a^	71	Man	Yes	Primary	10.61	No	No	Pro	17
AC6287	1	*NUDT3::MAP4*^a^	48	Man	Yes	Primary	27.86	No	NA	Cla	18
AA6367	2	*ZMPSTE24::CACNA1D*^a^*ADD2::C2orf42*^a^	53	Woman	No	Primary	4.53	No	No	Cla	18
AC6255	1	*AVIL::CPM*^a^	77	Man	Yes	Primary	21.36	No	No	Mes	18
AC6246	1	*TSFM::KIF5A*^a^	65	Woman	No	Primary	12.98	No	No	Pro	0
AC6253	1	*PDIA5::IQCB1*^a^	73	Man	Yes	Primary	9.69	No	No	Mes	23
AC6237	1	*LAMA5::PSMD3*^a^	71	Woman	Yes	Primary	4.30	No	No	Cla	18
AC6281	1	*KIF5A::AVIL*^a^	79	Man	Yes	Primary	2.79	No	No	Pro	9
AC6282	1	*WSB1::SEZ6*^a^	64	Woman	No	Primary	8.74	No	No	Pro	18
AA6373	1	*PIK3CB::EPHB1*^b^	54	Man	No	Primary	24.15	No	No	Pro	18
AA6366	1	*TBK1::TMPRSS12*^b^	79	Man	No	Primary	16.30	No	No	Mes	18
AC6276	1	*CREB5::ABCA13*^b^	78	Man	Yes	Primary	8.15	No	No	Cla	18
AA6380	4	*AGAP2::KIF5A*^c^ *EGFR::R3HDM2*^c^ *USP22::TMC3*^c^ *KCNMB4::C*^a^	61	Man	Yes	Primary	36.50	No	No	Pro	22
AC0344	1	*CEP78::GNAQ*^c^	57	Man	Yes	Primary	26.18	No	No	Cla	18
AA6364	1	*EGFR::SEPTIN14*^d^	55	Woman	Yes	Primary	42.55	NA	No	NA	NA
AC0438	1	*EGFR::SEPTIN14*^d^	62	Woman	Yes	Primary	21.13	No	No	Cla	18
AC0364	3	*CLIC4::SRRM1*^d^ *DPYSL3::JAKMIP2*^c^ *ABL1::SZRD1*^c^	62	Woman	No	Secondary	12.65	No	No	Cla	18
AC6239	1	*CAPZA2::MET*^d^	80	Man	No	Primary	1.51	No	No	Pro	9
AA6397	1	*FGFR3::TACC3*^d^	63	Man	No	Primary	32.89	No	No	Cla	18
AC6283	1	*FGFR3::TACC3*^d^	70	Man	No	Primary	9.76	No	NA	Cla	18
AC2104	3	*FGFR3::TACC3*^d^ *CTDSP2-GLI1*^c^ *CTDSP2::INHBE*^a^	75	Man	Yes	Primary	30.82	No	No	Cla	18
AA6386	2	*SEC61G::EGFR*^d^ *CALD1-ADAM22*^a^	70	Woman	No	Primary	10.81	No	No	Pro	18

*IHC* immunohistochemistry, *Pro* proneural, *Cla* classical, *Mes* mesenchymal, *NA* not available.

^a^In-frame fusion that can produce a protein but that has not previously been described in cancer, does not involve an oncogene or tumor suppressor gene, and was not predicted to have oncogenic potential by Oncofuse.

^b^Fusion predicted by Oncofuse to have oncogenic potential.

^c^Fusion not previously described in cancer but involving an oncogene or tumor suppressor gene.

^d^Fusion previously described in cancer.

### Clinical characteristics of patients with gene fusions with oncogenic potential

Twenty-four patient samples harbored one or more of the 30 fusions categorized as having oncogenic potential (Table 4). Two tumor samples had two fusions, two had three fusions, and one had four fusions. When we compared the clinical characteristics of the patients whose tumors had one or more of these fusions, there was no correlation with patient age or MGMT methylation status. All patients except one were IDH wild-type. Two patients were secondary glioblastomas with a history of previous low-grade glioma that had been treated with surgery alone. Three patients were classified as TCGA mesenchymal subtype, one of whom was classified as IGS-23 subtype, while the remaining patients were TCGA classical or proneural.

Two fusions had previously been associated with glioblastoma: *FGFR3::TACC3* and *EGFR::SEPTIN14*. Three patients had the *FGFR3::TACC3* fusion, all of whom were men older than 63 years and one of whom had MGMT methylation. Two patients had the *EGFR::SEPTIN14* fusion, both of whom were women with MGMT methylation (Table 4). The remaining fusions with oncogenic potential were each found in only one patient; this low frequency precluded a validation by RT-PCR of these fusions.

There were no differences in overall survival between patients with no fusions, those with fusions with oncogenic potential, and those with non-oncogenic fusions (*p* = 0.59).

### Comparison of fusions in FFPE vs FF tumor tissue

Four patients had paired FFPE and FF tissue from the first surgery and four others had paired FFPE tissue from both the first and second surgery. More fusions were detected in FF than in FFPE tissue, but fusions with oncogenic potential were detected in both types of samples. *EGFR::SEPTIN14* was detected in both FFPE and FF samples from one patient; *CLIC4::SRRM1*, *ZMPSTE24::CACNA1D* and *ADD2::C2orf42* were detected in samples from another patient; and *TBK1::TMPRSS12* was detected in samples from a third patient. *FGFR3::TACC3* was detected in the FFPE sample from the first surgery but not in the sample from the second surgery.

## Discussion

In order to explore the role of gene fusions in glioblastoma, we have analyzed fusions by RNA-Seq in tumor samples from 139 newly diagnosed, uniformly treated glioblastoma patients. Since our RNA-Seq results provided a long list of gene fusions, we performed an in silico study to predict their oncogenic potential. We first eliminated the fusions previously described in healthy tissue and then selected those previously described in cancer, those involving oncogenes or TSGs, and those identified by Oncofuse^22^ as having oncogenic potential. We limited our selection to in-frame fusions that could produce a protein with a biological effect. We identified a final list of 30 gene fusions with oncogenic potential, which were present in glioblastoma samples from 24 of the 139 patients included in the study. We then examined the frequency of these fusions in our series of glioblastoma patients and their potential correlation with patient characteristics and outcome.

RNA-Seq is useful in the assessment of tumors as a method to detect druggable fusions^23^. However, it provides a multitude of data that do not necessarily have biological significance. Moreover, several of the fusions have not been properly validated individually in the tissue in question, probably because of the large amount of data obtained and the difficulty of identifying the fusions that are biologically meaningful.

Methods for the detection of gene fusions are constantly evolving and it is certain that new methods will become available in the future. We used several methods in our analyses. For example, we used STAR fusion^6^ but later ARRIBA^24^ became available. Nevertheless, a recent study has shown that both of these methods outperformed others and were equally accurate at detecting fusions^25^. In addition, we ran DEEPrior^26^ in parallel with Oncofuse^22^ but chose Oncofuse as the final method since we found that Oncofuse results were more reliable. We also considered using PEGASUS^27^ but at the time of our study, it used the old version of the human genome (hg19), which dates from 2014. Finally, another method, ChimerDriver, has just been reported this year^28^. This diversity of currently available and newly emerging platforms means that it will be necessary to carefully determine the best method to use in the future to detect gene fusions and to identify those with oncologic potential. Two fusions identified as having oncogenic potential in our study had previously been associated with glioblastoma: *FGFR3::TACC3* and *EGFR::SEPTIN14*. The *FGFR-TACC* fusion has been reported in 1.2–8.3% of glioblastomas^29,30^. The latest WHO classification of gliomas describes fusions that occur in IDH-wild-type glioblastoma at an estimated frequency of > 1% ^10^. *EGFR*, one of the most frequent genes involved in recurrent in-frame fusions, is commonly found fused to *SEPTIN14* or to *PSPH*, with a frequency of 4% and 2.2%, respectively, in glioblastoma^31^. In our series, *EGFR* was involved in fusions in 5% of patients but not all the fusions involving *EGFR* had oncogenic potential. In fact, in-frame fusions involving *EGFR* with oncogenic potential were only detected in four patients (2.8%): *EGFR::SEPTIN14* in two samples, *SEC61G::EGFR* in one, and *EGFR::R3HDM2* in one. Of these, only the *EGFR::SEPTIN14* fusion was a bona fide driver, as the *SEC61G::EGFR* and *EGFR::R3HDM2* fusion proteins would lack the EGFR tyrosine kinase domain. The remaining *EGFR* fusions detected would produce either a frameshift transcript or no transcript at all.

Other *EGFR* alterations are also frequent in glioblastoma, including *EGFR* amplification, the *EGFR*vIII mutation, and altered splicing and rearrangements^32,33^. In our study, *EGFR* amplification was detected by FISH in all cases with EGFR fusions except one (a case with the *EGFR::SEPTIN14* fusion where there was insufficient available tissue for FISH analysis) (data not shown). The co-occurrence of *EGFR* fusions with *EGFR* amplification and *EGFR* vIII (exon 2–7 deletion) has also been previously reported^34^. This “two-hit” alteration has been described for several oncogenes in different tumor types, and it has been suggested that these oncogenes would be dosage-sensitive, with amplification of a mutated copy further increasing tumor fitness^35^. This could be the case in our specimen with the co-occurrence of the *EGFR::SEPTIN14* fusion and *EGFR* amplification, but it would not explain the existence of putatively non-functional *EGFR* fusions in cases with *EGFR* amplification. However, previous studies in glioblastoma have reported an increase in DNA breaks near genes targeted by copy number gains, including *EGFR*^36^. Taking this into account, we can speculate that the non-functional *EGFR* fusions could be the by-product of localized genome instability and would thus have no significance in the biology of the tumor. Along these same lines, in our study, we have detected several non-productive gene fusions in the 12q region, another breakpoint-enriched region in glioblastoma.

Unfortunately, therapies targeting different alterations of *EGFR* have failed to confer survival benefit^37–41^, although these studies did not include *EGFR* fusions. Fusions involving the *NTRK* genes have also been reported in glioblastoma, but they are more common in pediatric populations^42^ and were not detected in our samples. Other fusions reported in glioblastoma, including *LANCL2::RP11-745C15.2*, *LANCL2::SEPTIN14*, and *PTPRZ1::MET* ^43,44^, were not detected in our samples, although the *CAPZA2::MET* fusion was detected in one sample (0.7%). At present, only some fusions previously detected in glioblastoma are potentially druggable: *ROS1* fusions^45^, *FGFR::TACC*^46^, *NTRK* fusions^12,14^, and *MET* fusions^47^.

In the present study, we have identified 30 fusions with oncogenic potential. Each of these fusions was detected in < 1% of cases. Therefore, although our intention was to validate by RT-PCR the fusions identified in our in silico study, their low frequency made it unreasonable to do so in our series of patients. Such a low frequency of potentially oncogenic gene fusions suggests that the detection of individual fusions by RT-PCR would be neither reasonable nor cost-effective and that RNA-Seq would thus be the best procedure for searching for targetable fusions. Moreover, we found no correlation with patient characteristics that could identify a patient as a potential holder of any specific fusion.

Nonetheless, although many of the fusions identified in our study have not yet been described in glioblastoma, several of them involve actionable gene alterations that have been successfully targeted in other cancers. Considering the rarity of specific gene fusions in glioblastoma, it is not feasible to conduct a clinical trial limited to this subset of patients. However, the implementation of NGS in the molecular characterization of tumors is helping to identify a constantly increasing number of molecular alterations that are present in small subsets of a plethora of tumor types. We therefore believe that the NGS analysis of glioblastoma may allow the inclusion of glioblastoma patients in exploratory basket trials of specific tumor-agnostic biomarkers, as has been done with other rare gene alterations^48^. Our in silico study to detect in-frame fusions with oncogenic potential thus provides a good starting point for the identification of fusions that may be relevant to the pathogenesis or treatment of glioblastoma.

## Methods

### Patients and study design

From 2004 to 2014, the GLIOCAT project^49,50^ collected clinical data from 432 consecutive glioblastoma patients from six institutions, all of whom had received the standard first-line treatment (surgery followed by radiotherapy with concurrent and adjuvant temozolomide). The pathological diagnosis was confirmed by pathologists according to WHO 2007 classification guidelines^51^ before patients were included in the project. Once selected for inclusion, each case was anonymized and given a number to identify it across all data.

The following data were recorded: age, sex, symptoms, tumor characteristics, radiological characteristics, type of surgery, post-surgical performance, Mini Mental Status Examination (MMSE) score, details of radiotherapy and temozolomide treatments and treatment at relapse, date of progression, subsequent treatments, date and status at last control, and date and status of death or last control alive. Once patients were included in the study, *MGMT* methylation status was determined if it had not previously been assessed.

This study was approved by the Institutional Review Board of the Hospital Germans Trias i Pujol (PI-14-016) and by the Ethics Committees of all the participating institutions and their biobanks and was conducted in accordance with the ethical standards as laid down in the 1964 Declaration of Helsinki and its later amendments. All patients or their representatives gave their informed consent.

### Tissue microarray (TMA) construction and immunohistochemical analyses (IHC)

TMAs were constructed using a Veridiam Tissue Array Instrument (El Cajon, Ca, USA), model VTA-100, using a 1-mm diameter needle. Consecutive 4-µmm-thick sections were obtained and hematoxylin–eosin staining was done in sections 1, 20, and 40 in order to evaluate the persistence of the tumor at each spot.

IDH1-R132H analysis was done with the Dianova Cat# DIA-H09, RRID:AB_2335716, antibody. Four cases with doubtful IHC were sequenced to assess IDH status.

### DNA extraction and assessment of MGMT methylation

DNA was extracted from two 15-µm sections of FFPEtissue using the QIAamp DNA Mini Kit (QIAGEN GmbH, Hilden, Germany), following the manufacturer's protocol. In cases with less than 50% of tumor cells, the tumor tissue was macrodissected manually. Then 500 ng of extracted DNA was subjected to bisulfite treatment using the EZ DNA Methylation-Gold Kit (Zymo Research Corporation, Irvine, CA). *MGMT* promoter methylation status was determined by methylation-specific PCR (MSP) as previously described^52^.

### RNA-Seq assessments

RNA extraction from FFPE and FFsamples was performed on five 15 µm-deep tissue sections using the RNeasy FFPE Kit (Qiagen, Hilden, Germany) according to the manufacturer’s recommendations. RNA quantity and purity were measured with the NanoDrop ND-1000 spectrophotometer (Thermo Fisher Scientific, Waltham, MA, USA) and the Qubit RNA HS Assay Kit (Invitrogen, Eugene, OR, USA). The highest-quality RNA samples were sent to the Centro Nacional de Análisis Genómico (CNAG-CRG, Barcelona, Spain) for analysis by RNA-Seq. Methods for assessing quantification, purity and quality of samples have been previously described^16,49^.

The libraries were sequenced on HiSeq2000 (Illumina) in paired-end mode with a read length of 2 × 76 bp using TruSeq SBS Kit v4. Each sample was sequenced in a fraction of a sequencing v4 flow cell lane, following the manufacturer’s protocol. Image analysis, base calling and quality scoring of the run were processed using Real Time Analysis (RTA 1.18.66.3) software and followed by the generation of FASTQ sequence files by CASAVA.

### Classification of glioblastoma molecular subtypes

The TCGA classification of glioblastoma molecular subtypes^17,33,53^ was performed with the GlioVis portal^18^. The GlioVis glioblastoma TCGA cohort according to the ssGSEA method was selected as training dataset for both glioblastoma molecular subtype and Glioma CpG island methylator phenotype (G-CIMP) predictions. The IGS classification of glioblastoma molecular subtypes was done with the R clusterRepro package^17,19^, using the centroids for IGS0, IGS9, IGS16, IGS17, IGS18, IGS22 and IGS23 subtypes, as described by Gravendeel^19^ and used in several European series^54,55^.

### Identification of candidate gene fusions

Figure 2 shows the procedures and data bases used in the present study to select the fusions with oncogenic potential. STAR-Fusion (https://github.com/STAR-Fusion/STAR-Fusion/tree/STAR-Fusion-v1.9.0)^6^ was used to detect fusion genes based on discordant read alignments. Predicted fusions were further validated with FusionInspector in “validate” mode, which re-aligns the reads to a reference containing the genome and the fusion-gene contigs identified in the former step. Candidate fusions were annotated according to prior knowledge of fusion transcripts relevant to cancer biology (or previously observed in normal samples and thus less likely to have oncogenic potential) and assessed for the impact of the predicted fusion event on coding regions, indicating whether the fusion was in-frame or frame-shifted, along with combinations of domains expected to exist in the putative chimeric protein.

### Selection of fusions with oncogenic potential

We then used FusionHub^20^ (https://fusionhub.demopersistent.com/), which provides information from 28 public fusion and gene databases, and other data bases in the literature^56^ (Fig. 2). We first eliminated fusions previously described in healthy tissue. We then identified fusions previously described in cancers, including gliomas, and looked at whether any of the genes in the fusions was known to be fused with other genes in cancers. Finally, we explored whether either gene had been identified as an oncogene or TSG or had been associated with cancer.

Next, we selected only in-frame fusions, which could produce a protein with biological effect, and manually verified the break-points with Integrative Genomics Viewer (IGV, version 2.9.4) using the reference sequence hg38^21^. We reviewed the exons of each gene involved in the fusion as well as the amino acids with respect to the reference sequence.

To predict the oncogenic potential of each fusion, we ran DEEPrior^26^ and Oncofuse^22^. We found that DEEPrior did not predict an oncodriver role for the known oncogenic fusion *FGFR3-TACC3*, which led us to choose Oncofuse (www.unav.es/genetica/oncofuse.html) for our analysis. Oncofuse provides information on the Bayesian probability of a fusion being a driver (or class 1), with a higher value indicating a higher probability, or a passenger (or class 0) by giving a Bonferroni-corrected P-value that does not take into account whether the fusion is in-frame when calculating the *P*-value. We set the probability of a fusion being a driver at *P* > 0.75 and the P-value for it being a passenger at *P* < 0.05.

These procedures provided us with a final list of fusions with probable oncogenic potential in glioblastoma and allowed us to classify them into four categories: (1) fusions that had previously been described in cancer; (2) fusions that had not been described in cancer but that involve genes previously described as oncogenes or TSGs; (3) fusions that did not meet the above conditions but had a high Oncofuse probability of having oncogenic potential; and (4) fusions that did not meet any of these conditions but produce a protein product. We then looked at the incidence of the selected fusions in our sample set. We compared the results obtained in FFPE and paired FF tissue from the same patient and compared the results found in samples obtained at initial surgery and those obtained at relapse.

## Supplementary Information

Supplementary Information 1.

Supplementary Information 2.

Supplementary Information 3.

## Data Availability

The datasets generated during the current study are available from the corresponding author on reasonable request. Molecular data underlying the findings described in the manuscript are fully available without restriction from the Bioproject Sequence Read Archive: https://www.ncbi.nlm.nih.gov/sra/PRJNA833243 and http://www.ncbi.nlm.nih.gov/bioproject/613395.
