# Supplementary material for: In silico validation of RNA-Seq results can identify gene fusions with oncogenic potential in glioblastoma
Source: Sci Rep. 2022 Aug 24;12:14439. doi: 10.1038/s41598-022-18608-8 (PMC9402576; doi:10.1038/s41598-022-18608-8)
Supplement: Supplementary file 3 — Supplementary Information 3. [file 41598_2022_18608_MOESM3_ESM.docx]

***In silico* validation of RNA-Seq results can identify gene fusions with oncogenic potential in glioblastoma**

Ainhoa Hernandez, Ana Maria Muñoz-Marmol, Anna Esteve-Codina,Francesc Alameda, Cristina Carrato, Estela Pineda, Oriol Arpí-Llucià, Maria Martinez-García, Mar Mallo, Marta Gut, Sonia del Barco, Oscar Gallego, Marc Dabad, Carlos Mesia, Beatriz Bellosillo, Marta Domenech, Noemí Vidal, Iban Aldecoa, Nuria de la Iglesia, Carmen Balana

**Supplementary Information**

**Supplementary Table S1.** 103 fusions detected by RNA-Seq in 61 of 139 glioblastoma patients

**Supplementary Table S2.** Genes involved in 103 gene fusions identified in 61/139 glioblastoma

tumor samples

**Supplementary Table S3.** Classification of genes involved in 103 gene fusions identified in 61

glioblastoma samples

**Supplementary Table S1.** 103 fusions detected by RNA-Seq in 61 of 139 glioblastoma patients.

**Fusion**

*RP11-444D3.1::SOX5 RP11-384F7.2::LSAMP RP1-34H18.1::NAV3 RP1-161N10.1::TENM1 FGFR3::TACC3 PFKFB3::RP11-563J2.2 CLIC4::SRRM1 EGFR::SEPT14 LANCL2::RP11-745C15.2 ABL1::SZRD1 ACVR1B::SCAF11 ADD2::C2orf42 AGAP2::KIF5A ANTXR1::LINC01122 ARF4::GFRA2 ATP6V0A1::RALGAPA2 AVIL::CPM*

*BBS9::CREB5 BEST3::EGFR C1R::RASSF9 C3orf62::PBRM1 CALD1::ADAM22 CAMK2B::PSPH CAPZA2::MET CCT6A::PDE1C CD163L1::TSPAN31 CDK6::RP11-745C15.2 CELF2::ULK4 CEP78::GNAQ CNOT1::FAM118B CNOT2::LRP1 CNOT2::RBMS2 CREB5::ABCA13 CRY1::SCARB1 CTDSP2::FRS2 CTDSP2::GLI1 CTDSP2::INHBE CTDSP2::SEC61G DCTN2::RAB3IP*

*DPYSL3::JAKMIP2*

**Times detected**

29 13 10 4 3 3 2 2 2 1 1 1 1 1 1 1 1 1 1 1 1 1 1 1 1 1 1 1 1 1 1 1 1 1 1 1 1 1 1

1

**% of all times that fusions were detected (N=162)**

17.90 8.02 6.17 2.47 1.85 1.85 1.23 1.23 1.23 0.62 0.62 0.62 0.62 0.62 0.62 0.62 0.62 0.62 0.62 0.62 0.62 0.62 0.62 0.62 0.62 0.62 0.62 0.62 0.62 0.62 0.62 0.62 0.62 0.62 0.62 0.62 0.62 0.62 0.62

0.62

**% of all samples with fusions (N=61)**

47.5 21.3 16.4 6.6 4.9 4.9 3.3 3.3 3.3

1.6 1.6

1.6 1.6 1.6 1.6 1.6 1.6 1.6 1.6 1.6 1.6 1.6 1.6 1.6 1.6 1.6 1.6 1.6 1.6 1.6 1.6 1.6 1.6 1.6 1.6 1.6 1.6 1.6 1.6

1.6

**% of all samples analyzed (N=139)**

20.9 9.4 7.2 2.9 2.2 2.2 1.4 1.4 1.4

0.7 0.7

0.7 0.7 0.7 0.7 0.7 0.7 0.7 0.7 0.7 0.7 0.7 0.7 0.7 0.7 0.7 0.7 0.7 0.7 0.7 0.7 0.7 0.7 0.7 0.7 0.7 0.7 0.7 0.7

0.7

*EGFR::R3HDM2* 1 0.62 1.6 0.7 *EGFR::RP11-745C15.2* 1 0.62 1.6 0.7 *FRS2::KIF5A* 1 0.62 1.6 0.7 *GNAQ::CEP78* 1 0.62 1.6 0.7 *GTF3C4::LINC00698* 1 0.62 1.6 0.7 *HMGA2::LLPH* 1 0.62 1.6 0.7 *HRH1::EGFR-AS1* 1 0.62 1.6 0.7 *IFT74::DENND4C* 1 0.62 1.6 0.7 *ISY1::MARS* 1 0.62 1.6 0.7 *JAZF1::SEPT7P5* 1 0.62 1.6 0.7 *KCNMB4::CNOT2* 1 0.62 1.6 0.7 *KIF5A::AVIL* 1 0.62 1.6 0.7 *LAMA5::PSMD3* 1 0.62 1.6 0.7 *LANCL2::VOPP1* 1 0.62 1.6 0.7 *LINC00907::RP11-116O1* 1 0.62 1.6 0.7 *LINC00971::ROBO2* 1 0.62 1.6 0.7 *LRP1::CTDSP2* 1 0.62 1.6 0.7 *MALAT1::EGFR* 1 0.62 1.6 0.7 *MAPKAPK2::SOX13* 1 0.62 1.6 0.7 *MBD6::ARHGAP9* 1 0.62 1.6 0.7 *MITF::ST18* 1 0.62 1.6 0.7 *NFYA::RP3-340B19.3* 1 0.62 1.6 0.7 *NUDT3::MAP4* 1 0.62 1.6 0.7 *NUP214::TMOD1* 1 0.62 1.6 0.7 *OCIAD1::FRYL* 1 0.62 1.6 0.7 *PBX3::RPL35* 1 0.62 1.6 0.7 *PDE1C::CEP41* 1 0.62 1.6 0.7 *PDIA5::IQCB1* 1 0.62 1.6 0.7 *PHC3::EGFEM1P* 1 0.62 1.6 0.7 *PID1::DNER* 1 0.62 1.6 0.7 *PIK3CB::EPHB1* 1 0.62 1.6 0.7 *PTPRB::LRP1* 1 0.62 1.6 0.7 *R3HDM2::AVIL* 1 0.62 1.6 0.7 *RAB3IP::BEST3* 1 0.62 1.6 0.7 *RALA::SUGCT* 1 0.62 1.6 0.7 *RAP1B::ARHGEF25* 1 0.62 1.6 0.7 *RAP1B::LRP1* 1 0.62 1.6 0.7 *REEP5::EPB41L4A* 1 0.62 1.6 0.7 *RERE::PSMD6* 1 0.62 1.6 0.7 *RNF165::LINC00907* 1 0.62 1.6 0.7 *RNF41::RAB21* 1 0.62 1.6 0.7 *RP11-320P7.2::CNOT2* 1 0.62 1.6 0.7 *RP11-620J15.2::CD163L* 1 0.62 1.6 0.7 *SCARB1::KRT8P3* 1 0.62 1.6 0.7 *SEC61G::EGFR* 1 0.62 1.6 0.7

*SLC31A1::PRKAR2A* 1 0.62 1.6 0.7 *SLC35E3::EGFR* 1 0.62 1.6 0.7 *SLC4A8::TMEM132D* 1 0.62 1.6 0.7 *STAT3::CFAP61* 1 0.62 1.6 0.7 *SUCLG2::GLYCTK* 1 0.62 1.6 0.7 *SYNGAP1::DLG1* 1 0.62 1.6 0.7 *TBK1::TMPRSS12* 1 0.62 1.6 0.7 *TIGAR::MED21* 1 0.62 1.6 0.7 *TMCO3::USP24P1* 1 0.62 1.6 0.7 *TMEM132D::PPFIA2* 1 0.62 1.6 0.7 *TSFM::CCDC60* 1 0.62 1.6 0.7 *TSFM::KIF5A* 1 0.62 1.6 0.7 *USP22::TMC3* 1 0.62 1.6 0.7 *VPS53::VWDE* 1 0.62 1.6 0.7 *WIPI1::RP1-193H18.3* 1 0.62 1.6 0.7 *WSB1::SEZ6* 1 0.62 1.6 0.7 *XRCC5::LINC01614* 1 0.62 1.6 0.7 *ZMPSTE24::CACNA1D* 1 0.62 1.6 0.7

**Supplementary Table S2.** Genes involved in 103 gene fusions identified in 61/139 glioblastoma tumor samples.

**Supplementary Table S2A.** The number of samples in which a fusion involving the gene was identified.

**Gene**

*RP11-444D3.1 SOX5 LSAMP*

*RP11-384F7.2 NAV3*

*RP1-34H18.1 EGFR CTDSP2 CNOT2 KIF5A LRP1*

*RP1-161N10.1 RP11-745C15.2 TENM1 AVIL FGFR3 LANCL2 PFKFB3J2.2*

*RP11-563J2.2 TACC3 BEST3 CD163L1 CEP78 CLIC4 CREB5 FRS2 GNAQ LINC00907 PDE1C R3HDM2 RAB3IP RAP1B SCARB1 SEC61G SEPT14 SRRM1*

*TMEM132D*

*TSFM*

**No. samples**

29 29 13 13 10 10 8 5 4 4 4 4 4 4 3 3 3 3 3 3 2 2 2 2 2 2 2 2 2 2 2 2 2 2 2 2 2

2

**% of all samples analyzed (N=139)**

20.86 20.86 9.35 9.35 7.19 7.19 5.76 3.6 2.88 2.88 2.88 2.88 2.88 2.88 2.16 2.16 2.16 2.16 2.16 2.16 1.44 1.44 1.44 1.44 1.44 1.44 1.44 1.44 1.44 1.44 1.44 1.44 1.44 1.44 1.44 1.44 1.44

1.44

**% of all samples with fusions (N=61)**

47.54 47.54 21.31 21.31 16.39 16.39 13.11 8.2 6.56 6.56 6.56 6.56 6.56 6.56 4.92 4.92 4.92 4.92 4.92 4.92 3.28 3.28 3.28 3.28 3.28 3.28 3.28 3.28 3.28 3.28 3.28 3.28 3.28 3.28 3.28 3.28 3.28

3.28

*ABCA13* 1 0.72 1.64 *ABL1* 1 0.72 1.64 *ACVR1B* 1 0.72 1.64 *ADAM22* 1 0.72 1.64 *ADD2* 1 0.72 1.64 *AGAP2* 1 0.72 1.64 *ANTXR1* 1 0.72 1.64 *ARF4* 1 0.72 1.64 *ARHGAP9* 1 0.72 1.64 *ARHGEF25* 1 0.72 1.64 *ATP6V0A1* 1 0.72 1.64 *BBS9* 1 0.72 1.64 *C1R* 1 0.72 1.64 *C2orf42* 1 0.72 1.64 *C3orf62* 1 0.72 1.64 *CACNA1D* 1 0.72 1.64 *CALD1* 1 0.72 1.64 *CAMK2B* 1 0.72 1.64 *CAPZA2* 1 0.72 1.64 *CCDC60* 1 0.72 1.64 *CCT6A* 1 0.72 1.64 *CDK6* 1 0.72 1.64 *CELF2* 1 0.72 1.64 *CEP41* 1 0.72 1.64 *CFAP61* 1 0.72 1.64 *CNOT1* 1 0.72 1.64 *CPM* 1 0.72 1.64 *CRY1* 1 0.72 1.64 *DCTN2* 1 0.72 1.64 *DENND4C* 1 0.72 1.64 *DLG1* 1 0.72 1.64 *DNER* 1 0.72 1.64 *DPYSL3* 1 0.72 1.64 *EGFEM1P* 1 0.72 1.64 *EGFR-AS1* 1 0.72 1.64 *EPB41L4A* 1 0.72 1.64 *EPHB1* 1 0.72 1.64 *FAM118B* 1 0.72 1.64 *FRYL* 1 0.72 1.64 *GFRA2* 1 0.72 1.64 *GLI1* 1 0.72 1.64 *GLYCTK* 1 0.72 1.64 *GTF3C4* 1 0.72 1.64 *HMGA2* 1 0.72 1.64 *HRH1* 1 0.72 1.64

*IFT74* 1 0.72 1.64 *INHBE* 1 0.72 1.64 *IQCB1* 1 0.72 1.64 *ISY1* 1 0.72 1.64 *JAKMIP2* 1 0.72 1.64 *JAZF1* 1 0.72 1.64 *KCNMB4* 1 0.72 1.64 *KRT8P3* 1 0.72 1.64 *LAMA5* 1 0.72 1.64 *LINC00698* 1 0.72 1.64 *LINC00971* 1 0.72 1.64 *LINC01122* 1 0.72 1.64 *LINC01614* 1 0.72 1.64 *LLPH* 1 0.72 1.64 *MALAT1* 1 0.72 1.64 *MAP4* 1 0.72 1.64 *MAPKAPK2* 1 0.72 1.64 *MARS* 1 0.72 1.64 *MBD6* 1 0.72 1.64 *MED21* 1 0.72 1.64 *MET* 1 0.72 1.64 *MITF* 1 0.72 1.64 *NFYA* 1 0.72 1.64 *NUDT3* 1 0.72 1.64 *NUP214* 1 0.72 1.64 *OCIAD1* 1 0.72 1.64 *PBRM1* 1 0.72 1.64 *PBX3* 1 0.72 1.64 *PDIA5* 1 0.72 1.64 *PHC3* 1 0.72 1.64 *PID1* 1 0.72 1.64 *PIK3CB* 1 0.72 1.64 *PPFIA2* 1 0.72 1.64 *PRKAR2A* 1 0.72 1.64 *PSMD3* 1 0.72 1.64 *PSMD6* 1 0.72 1.64 *PSPH* 1 0.72 1.64 *PTPRB* 1 0.72 1.64 *RAB21* 1 0.72 1.64 *RALA* 1 0.72 1.64 *RALGAPA2* 1 0.72 1.64 *RASSF9* 1 0.72 1.64 *RBMS2* 1 0.72 1.64 *REEP5* 1 0.72 1.64 *RERE0* 1 0.72 1.64

*RNF165* 1 0.72 1.64 *RNF41* 1 0.72 1.64 *ROBO2* 1 0.72 1.64 *RP1-193H18.3* 1 0.72 1.64 *RP11-116O18.3* 1 0.72 1.64 *RP11-320P7.2* 1 0.72 1.64 *RP11-620J15.2* 1 0.72 1.64 *RP3-340B19.3* 1 0.72 1.64 *RPL35* 1 0.72 1.64 *SCAF11* 1 0.72 1.64 *SEPT7P5* 1 0.72 1.64 *SEZ6* 1 0.72 1.64 *SLC31A1* 1 0.72 1.64 *SLC35E3* 1 0.72 1.64 *SLC4A8* 1 0.72 1.64 *SOX13* 1 0.72 1.64 *ST18* 1 0.72 1.64 *STAT3* 1 0.72 1.64 *SUCLG2* 1 0.72 1.64 *SUGCT* 1 0.72 1.64 *SYNGAP1* 1 0.72 1.64 *SZRD1* 1 0.72 1.64 *TBK1* 1 0.72 1.64 *TIGAR* 1 0.72 1.64 *TMC3* 1 0.72 1.64 *TMCO3* 1 0.72 1.64 *TMOD1* 1 0.72 1.64 *TMPRSS12* 1 0.72 1.64 *TSPAN31* 1 0.72 1.64 *ULK4* 1 0.72 1.64 *USP22* 1 0.72 1.64 *USP24P1* 1 0.72 1.64 *VOPP1* 1 0.72 1.64 *VPS53* 1 0.72 1.64 *VWDE* 1 0.72 1.64 *WIPI1* 1 0.72 1.64 *WSB1* 1 0.72 1.64 *XRCC5* 1 0.72 1.64 *ZMPSTE24* 1 0.72 1.64

**Supplementary Table S2B.** The number of fusions in which the gene was involved.

**Gene**

*EGFR CTDSP2 CNOT2 KIF5A LRP1 AVIL*

*RP11-745C15.2 BEST3 CD163L1 CEP78 CREB5 FRS2 GNAQ LANCL2 LINC00907 PDE1C R3HDM2 RAB3IP RAP1B SCARB1 SEC61G*

*TMEM132D TSFM ABCA13 ABL1 ACVR1B ADAM22 ADD2 AGAP2 ANTXR1 ARF4 ARHGAP9 ARHGEF25 ATP6V0A1 BBS9 C1R C2orf42 C3orf62*

*CACNA1D CALD1*

*CAMK2B*

**No. fusions**

7 5 4 4 4 3 3 2 2 2 2 2 2 2 2 2 2 2 2 2 2 2 2 1 1 1 1 1 1 1 1 1 1 1 1 1 1 1 1 1

1

**% of all samples analyzed (N=139)**

5.04 3.6 2.88 2.88 2.88 2.16 2.16 1.44 1.44 1.44 1.44 1.44 1.44 1.44 1.44 1.44 1.44 1.44 1.44 1.44 1.44 1.44 1.44 0.72 0.72 0.72 0.72 0.72 0.72 0.72 0.72 0.72 0.72 0.72 0.72 0.72 0.72 0.72 0.72 0.72

0.72

**% of all samples with fusions (N=61)**

11.48 8.2 6.56 6.56 6.56 4.92 4.92 3.28 3.28 3.28 3.28 3.28 3.28 3.28 3.28 3.28 3.28 3.28 3.28 3.28 3.28 3.28 3.28 1.64 1.64 1.64 1.64 1.64 1.64 1.64 1.64 1.64 1.64 1.64 1.64 1.64 1.64 1.64 1.64 1.64

1.64

*CAPZA2* 1 0.72 1.64 *CCDC60* 1 0.72 1.64 *CCT6A* 1 0.72 1.64 *CDK6* 1 0.72 1.64 *CELF2* 1 0.72 1.64 *CEP41* 1 0.72 1.64 *CFAP61* 1 0.72 1.64 *CLIC4* 1 0.72 1.64 *CNOT1* 1 0.72 1.64 *CPM* 1 0.72 1.64 *CRY1* 1 0.72 1.64 *DCTN2* 1 0.72 1.64 *DENND4C* 1 0.72 1.64 *DLG1* 1 0.72 1.64 *DNER* 1 0.72 1.64 *DPYSL3* 1 0.72 1.64 *EGFEM1P* 1 0.72 1.64 *EGFR-AS1* 1 0.72 1.64 *EPB41L4A* 1 0.72 1.64 *EPHB1* 1 0.72 1.64 *FAM118B* 1 0.72 1.64 *FGFR3* 1 0.72 1.64 *FRYL* 1 0.72 1.64 *GFRA2* 1 0.72 1.64 *GLI1* 1 0.72 1.64 *GLYCTK* 1 0.72 1.64 *GTF3C4* 1 0.72 1.64 *HMGA2* 1 0.72 1.64 *HRH1* 1 0.72 1.64 *IFT74* 1 0.72 1.64 *INHBE* 1 0.72 1.64 *IQCB1* 1 0.72 1.64 *ISY1* 1 0.72 1.64 *JAKMIP2* 1 0.72 1.64 *JAZF1* 1 0.72 1.64 *KCNMB4* 1 0.72 1.64 *KRT8P3* 1 0.72 1.64 *LAMA5* 1 0.72 1.64 *LINC00698* 1 0.72 1.64 *LINC00971* 1 0.72 1.64 *LINC01122* 1 0.72 1.64 *LINC01614* 1 0.72 1.64 *LLPH* 1 0.72 1.64 *LSAMP* 1 0.72 1.64 *MALAT1* 1 0.72 1.64

*MAP4* 1 0.72 1.64 *MAPKAPK2* 1 0.72 1.64 *MARS* 1 0.72 1.64 *MBD6* 1 0.72 1.64 *MED21* 1 0.72 1.64 *MET* 1 0.72 1.64 *MITF* 1 0.72 1.64 *NAV3* 1 0.72 1.64 *NFYA* 1 0.72 1.64 *NUDT3* 1 0.72 1.64 *NUP214* 1 0.72 1.64 *OCIAD1* 1 0.72 1.64 *PBRM1* 1 0.72 1.64 *PBX3* 1 0.72 1.64 *PDIA5* 1 0.72 1.64 *PFKFB3J2.2* 1 0.72 1.64 *PHC3* 1 0.72 1.64 *PID1* 1 0.72 1.64 *PIK3CB* 1 0.72 1.64 *PPFIA2* 1 0.72 1.64 *PRKAR2A* 1 0.72 1.64 *PSMD3* 1 0.72 1.64 *PSMD6* 1 0.72 1.64 *PSPH* 1 0.72 1.64 *PTPRB* 1 0.72 1.64 *RAB21* 1 0.72 1.64 *RALA* 1 0.72 1.64 *RALGAPA2* 1 0.72 1.64 *RASSF9* 1 0.72 1.64 *RBMS2* 1 0.72 1.64 *REEP5* 1 0.72 1.64 *RERE* 1 0.72 1.64 *RNF165* 1 0.72 1.64 *RNF41* 1 0.72 1.64 *ROBO2* 1 0.72 1.64 *RP1-161N10.1* 1 0.72 1.64 *RP1-193H18.3* 1 0.72 1.64 *RP1-34H18.1* 1 0.72 1.64 *RP11-116O18.3* 1 0.72 1.64 *RP11-320P7.2* 1 0.72 1.64 *RP11-384F7.2* 1 0.72 1.64 *RP11-444D3.1* 1 0.72 1.64 *RP11-563J2.2* 1 0.72 1.64 *RP11-620J15.2* 1 0.72 1.64 *RP3-340B19.3* 1 0.72 1.64

*RPL35* 1 0.72 1.64 *SCAF11* 1 0.72 1.64 *SEPT14* 1 0.72 1.64 *SEPT7P5* 1 0.72 1.64 *SEZ6* 1 0.72 1.64 *SLC31A1* 1 0.72 1.64 *SLC35E3* 1 0.72 1.64 *SLC4A8* 1 0.72 1.64 *SOX13* 1 0.72 1.64 *SOX5* 1 0.72 1.64 *SRRM1* 1 0.72 1.64 *ST18* 1 0.72 1.64 *STAT3* 1 0.72 1.64 *SUCLG2* 1 0.72 1.64 *SUGCT* 1 0.72 1.64 *SYNGAP1* 1 0.72 1.64 *SZRD1* 1 0.72 1.64 *TACC3* 1 0.72 1.64 *TBK1* 1 0.72 1.64 *TENM1* 1 0.72 1.64 *TIGAR* 1 0.72 1.64 *TMC3* 1 0.72 1.64 *TMCO3* 1 0.72 1.64 *TMOD1* 1 0.72 1.64 *TMPRSS12* 1 0.72 1.64 *TSPAN31* 1 0.72 1.64 *ULK4* 1 0.72 1.64 *USP22* 1 0.72 1.64 *USP24P1* 1 0.72 1.64 *VOPP1* 1 0.72 1.64 *VPS53* 1 0.72 1.64 *VWDE* 1 0.72 1.64 *WIPI1* 1 0.72 1.64 *WSB1* 1 0.72 1.64 *XRCC5* 1 0.72 1.64 *ZMPSTE24* 1 0.72 1.64

**Supplementary Table S3.** Classification of genes involved in 103 gene fusions identified in 61 glioblastoma samples.

**Supplementary Table S3A.** Genes that are known oncogenes or tumor suppressor genes (TSGs) (N=20)

**Gene** **Oncogene or TSG**

*PBRM1* Oncogene *FGFR3* Oncogene *GNAQ* Oncogene *MALAT1* Oncogene *MITF* Oncogene *RERE* Oncogene *LSAMP* Possible TSG *VPS53* Possible TSG *XRCC5* Possible TSG *CDK6* Oncogene or TSG *GLI1* Oncogene or TSG *STAT3* Oncogene or TSG *USP22* Oncogene or TSG *ABL1* Oncogene or TSG *AGAP2* Oncogene or TSG *EGFR* Oncogene or TSG *HMGA2* Oncogene or TSG *JAZF1* Oncogene or TSG

*MET* Oncogene or TSG

**Cancer-related?**

Yes Yes Yes Yes Yes Yes No No No Yes Yes Yes No Yes Yes Yes Yes Yes

Yes

**In fusions with other genes in gliomas?**

LG

With TACC3 in glioma No No No LG LG LG LG No

LG & GB No No No GB

LG & GB GB

LG & GB

GB

**Possible TSG?**

Yes Yes Yes Yes Yes Yes

Yes

*NUP214* Oncogene or TSG Yes LG LG, low-grade glioma; GB, glioblastoma

**Supplementary Table S3B.** Genes associated with cancer but not known oncogenes or TSGs (N=16)

**Gene**

*ACVR1B ARHGAP9 CAMK2B CLIC4 DLG1 FRYL*

*INHBE*

**Cancer-related?**

Yes Yes Yes Yes Yes Yes

Yes

**In fusions with other genes in gliomas?**

No No No GB No No

No

**Gene**

*MAPKAPK2 MARS PBX3 RALA RAP1B SCARB1*

*SOX5*

**Cancer-related?**

Yes Yes Yes Yes Yes Yes

Yes

**In fusions with other genes in gliomas?**

No GB No GB GB LG

LG

*MAP4* Yes LG *TACC3* Yes With FGFR3 in glioma LG, low-grade glioma; GB, glioblastoma

**Supplementary Table S3C.** Genes that are not known oncogenes or TSGs and are not associated with cancer but are involved in fusions with other genes in gliomas (N=32)

**Gene**

*CCDC60 CCT6A CNOT2 DCTN2 EPHB1 GTF3C4 IFT74 ISY1 PSMD3 PTPRB RNF165 SLC31A1 TMC3*

*TMEM132D*

*ULK4*

**Cancer-related?**

No No No No No No No No No No No No No No

No

**In fusions with other genes in gliomas?**

LG LG LG LG LG LG LG LG LG LG LG LG LG LG

LG

**Gene**

*AVIL CPM CREB5 LANCL2 LRP1 PPFIA2 R3HDM2 SLC35E3 SOX13 TSPAN31 WSB1 CTDSP2 KIF5A SEPTIN14*

*TSFM*

**Cancer-related?**

No No No No No No No No No No No No No No

No

**In fusions with other genes in gliomas?**

GB GB GB GB GB GB GB GB GB GB GB

LG & GB LG & GB LG & GB

LG & GB

*ATP6V0A1* No GB *FRS2* No GB LG, low-grade glioma; GB, glioblastoma

**Supplementary Table S3D.** Genes that are not oncogenes or TSGs, have not previously been described in cancer, and are not involved in fusions in gliomas (N=99)

*ABCA13 ADAM22 ADD2 ANTXR1 ARF4 ARHGEF25 BBS9 BEST3 C1R C2orf42 C3orf62 CACNA1D CALD1 CAPZA2 CD163L1 CELF2*

*CEP41*

*CEP78 CFAP61 CNOT1 CRY1 DENND4C DNER DPYSL3 EGFEM1P EGFR-AS1 EPB41L4A FAM118B GFRA2 GLYCTK HRH1 IQCB1*

*JAKMIP2*

*KCNMB4 KRT8P3 LAMA5 LINC00698 LINC00907 LINC00971 LINC01122 LINC01614 LLPH MBD6 MED21 NAV3 NFYA NUDT3 OCIAD1 PDE1C*

*PDIA5*

*PFKFB3J2.2 PHC3 PID1 PIK3CB PRKAR2A PSMD6 PSPH RAB21 RAB3IP*

*RALGAPA2 RASSF9 RBMS2 REEP5 RNF41 ROBO2*

*RP1-161N10.1*

*RP1-193H18.3 RP1-34H18.1 RP11-116O18.3 RP11-320P7.2 RP11-384F7.2 RP11-444D3.1 RP11-563J2.2 RP11-620J15.2 RP11-745C15.2 RP3-340B19.3 RPL35 SCAF11 SEC61G SEPT7P5 SEZ6 SLC4A8*

*SRRM1*

*ST18 SUCLG2 SUGCT SYNGAP1 SZRD1 TBK1 TENM1 TIGAR TMCO3 TMOD1 TMPRSS12 USP24P1 VOPP1 VWDE WIPI1*

*ZMPSTE24*
